# Projecting Cost Containment in the Operating Room Utilizing Incentivized Strategies to Reduce Healthcare Cost

**DOI:** 10.1097/pq9.0000000000000190

**Published:** 2019-06-22

**Authors:** Tanner Koppert, Dmitry Tumin, Joseph D. Tobias, Vidya T. Raman

**Affiliations:** From the * Department of Anesthesiology and Pain Medicine, Nationwide Children’s Hospital, Columbus, Ohio; † Department of Anesthesiology and Pain Medicine, The Ohio State University, College of Medicine, Columbus, Ohio

## Abstract

**Introduction:**

Streamlining healthcare in United States is of paramount concern while maintaining standards of quality and safety. Incentivizing change may be even more effective in driving such measures. At Nationwide Children’s Hospital, we incentivize cost savings directly to the healthcare team member. In this project, we evaluated a simple substitution of a buretrol for Y-type tubing based on weight rather than age cutoffs.

**Methods:**

This was deemed a quality improvement project and therefore exempt from Institutional Review Board approval. We obtained costs of Y-type tubing versus buretrols. We interrogated the electronic medical record to quantify case volume in the main operating room according to age and weight. We calculated our costs to compare our current practice of using buretrol fo age ≤ 12 years and the planned practice of using buretrol for weight < 20 kg.

**Results:**

We identified 28,875 children ages 0-12 (60% weight <20kg) and 6,301 children ages 13-18 (0.1% weight <20kg) undergoing procedures in the main operating rooms over a 1-year period.. A unit cost savings of $4.40 substituting Y-type tubing for a buretrol was determined. Transitioning from age-based to weight-based criteria for buretrol use was determined to potentially save $51,260 over the period reviewed.

**Conclusions:**

Simple changes can impact efficiency and cost in healthcare. It is important to consider incentivizing such measures to help drive these changes. In the future, with more incentivized measures, hopefully we can successfully make an impact of efficiency and cost of healthcare in United States without compromising safety or quality.

## INTRODUCTION

Streamlining healthcare in the United States, while maintaining high standards of quality and safety, is of paramount concern in operating rooms across the country. The Perioperative Surgical Home initiative by the American Society of Anesthesiologists has advocated improving efficiency and quality of care while decreasing cost.^1^ A 2011 study on the United States healthcare spending indicated that waste accounted for an estimated 47% of total spending.^2^ Currently, maintaining an inventory of even basic hospital supplies and medications can be challenging, and duplication of devices or supplies can increase costs. Hospitals are facing medical supply and medication shortages due to the disruption of distribution chains and manufacturing plants as a result of recent natural disasters, such as Hurricane Maria.^3,4^ With the physical demand going up, as seen with the caseload at Nationwide Children’s Hospital, and supply due to natural disasters going down, it causes uncontrollable shortages. Even without a sudden shortage of medical supplies, changes in practice may be indicated to reduce costs associated with procurement without adversely affecting patient safety.

Incentivizing quality improvement changes that lead to enhanced efficiency and cost savings may be effective in driving these changes. At Nationwide Children’s Hospital, we incentivized cost savings by providing educational stipends allowing a portion of cost savings to return to the healthcare provider who recommended the change. This stipend is awarded solely to the employee initiating the change. A review board consisting of 5 employees from the hospital determines the portion of the cost savings. Nationwide Children’s also provides transparency of material costs to employees to encourage creative solutions to decrease costs. As an example of this process, we provide information on how a simple substitution of intravenous tubing practice may affect healthcare costs.

## METHODS

Our standard practice was to use the buretrol-type intravenous tubing set for patients 12 years and younger of age. We evaluated a simple substitution of a Y-type tubing (Hospira Bloodset, Lake Forest, Ill.) instead of the Buretrolintravenous set (Clearlink, Baxter Deerfield, Ill.) based on weight rather than age cutoffs. This change yielded a unit cost savings of $4.40 per patient. We assessed the impact of switching to a policy in the operating room of reserving use of the Buretrol intravenous tubing to patients who weigh less than 20 kg as opposed to the current practice of those 12 years and younger of age. Anesthesia technicians use an electronic board with patient’s demographics, including weight, to assess case IV requirements.

## RESULTS

**Fig. 1.**
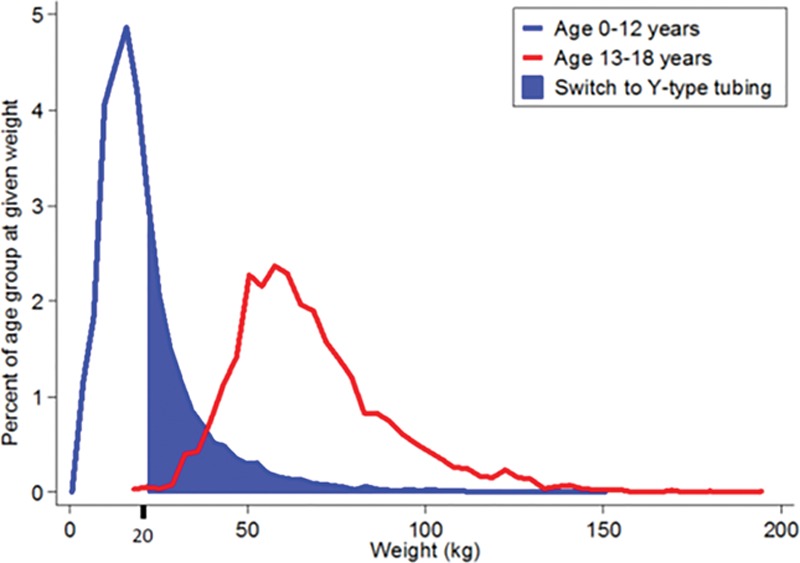
Distribution of patients according to the specific age group. The shaded blue area shows the patients who can likely safely be transitioned from the use of the Buretrol intravenous tubing set to the less expensive Y-tubing with a cost savings of approximately $4.40 per patient.

With a systematic review of the electronic medical record, we identified 28,875 children, age 0–12 years (60% weight < 20 kg) and 6,301 children, age 13–18 years (0.1% weight < 20 kg) undergoing procedures in the main operating rooms over one year in 2017. The distribution of weight by age group is illustrated in Figure 1. The shaded area indicates the patients who would be eligible for the proposed switch using the Y-tubing instead of the Buretrol intravenous tubing set. By transitioning from age-based to weight-based criteria for the use of the Buretrol intravenous tubing set, we postulate that we can save $51,260 over the period reviewed. See Table 1 for costs in 2017.

**Table 1.**
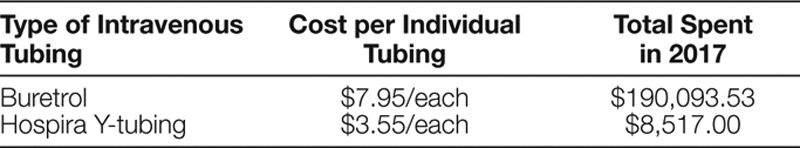
Actual Cost Spending in 2017 of Buretrol versus Hospira Y-tubing

## DISCUSSION

Providing cost data and incentives to healthcare providers can increase the opportunity for identifying potential savings by simple changes in routine practice, without compromising safety or quality. Previously, the Buretrol intravenous tubing set was used based on simple age criteria. The Buretrol intravenous tubing set is primarily used to avoid the risk of excessive fluid administration. As such concerns are the highest in the younger patients who weigh less, we decided that using a weight-based cutoff would be more appropriate given the significant variability in weight across children of the same age.

The previous use of an age cutoff was related to the ease of having the patient’s age on the operating room schedule, which would facilitate the preparation of intravenous fluids by the perioperative team. However, due to Joint Commission requirements and the implementation of a uniform electronic medical record across the hospital, including the operating room, we can change our practice. Easily obtainable weight information from the electronic record and new guidelines allow for more selective use of the Buretrol intravenous tubing set and increased use of the Y-tubing. Although communication remains essential in determining the type of tubing to use, this incentivized substitution has decreased the financial impact of the overusing the Buretrol intravenous tubing set. Although at the time of this letter, we have yet to introduce the change, we have thought of potential outcome balances and unintended consequences associated with making this change. One is regarding the actual rates of fluid given to the patient. The buterol intravenous tubing self-limits to 150 ml no matter size of the fluid bag placed and the rate of the droplet is more challenging to compute (60 drops equaling 1 ml) and can vary based on size vein cannulated and height of buterol line placement. However, the Y-tubing can give 500 ml with 22 compressions (20 drops is equal to 7 ml). The Y-tubing provides seven times the amount of fluid at a third of the rate. This factor can be an issue eliminating the Buretrol intravenous tubing for our small patients. However, due to the huge weight variations that present in our patients, from <1 to >200 kg, it may not be practical to eliminate buterols completely. There exists no real difference in how we administer medications through the 2 different fluid sets. Medications that need rate limiting are administered via an infusion pump and a secondary line attached to the primary tubing. There is a small difference in the setup. The buterol does take a few more minutes to run fluid through due to smaller drip chamber and increase the risk of entraining air bubbles. However, we generally set up approximately 100 IV setups a day of which Buretrols currently account for about half of the setups. A decrease in Buretrol use could have unintended consequences by altering cost savings that result from volume-based negotiated rates of supplies adversely effecting supplier and supply chain costs. However, these consequences will not truly be known until change effected.

Incentivizing employees to create cost-effective solutions are great ways to cut hospital costs as seen in this model, where the estimated cost savings are used to determine the percentage of compensation to the employee. Simple innovations like this may provide significant healthcare savings without compromising patient care.

## DISCLOSURE

The authors have no financial interest to declare in relation to the content of this article.
